# The Combination Therapy of Dissolution Using Carbonated Liquid and Endoscopic Procedure for Bezoars: Pragmatical and Clinical Review

**DOI:** 10.1155/2016/7456242

**Published:** 2016-08-23

**Authors:** Kohei Ogawa, Kenya Kamimura, Ken-ichi Mizuno, Yoko Shinagawa, Yuji Kobayashi, Hiroyuki Abe, Yukari Watanabe, Shunsaku Takahashi, Kazunao Hayashi, Junji Yokoyama, Manabu Takeuchi, Masaaki Kobayashi, Satoshi Yamagiwa, Yuichi Sato, Shuji Terai

**Affiliations:** Division of Gastroenterology and Hepatology, Graduate School of Medical Dental Sciences, Niigata University, 1-757 Asahimachi-dori, Chuo-ku, Niigata 951-8510, Japan

## Abstract

Bezoars are relatively rare foreign bodies of gastrointestinal tract and often cause ileus and ulcerative lesions in the stomach and subsequent bleeding and perforation due to their size and stiffness. Therefore, the removal of bezoars is essential and recent development of devices, the endoscopic removal procedure, is often applied. However, due to their stiffness, simple endoscopic removal failed in not a few cases, and surgical removal has also been used. Recently, the efficacy of a combination therapy of endoscopic procedure and dissolution using carbonated liquid has been reported. To develop the safe and effective removal procedure, we carefully reviewed a total of 55 reported cases in this study including our 3 additional cases, successfully treated with dissolution with endoscopic fragmentation. In summary, the data showed the efficiency in the combination therapy, treating the larger size of bezoar and reducing the length of hospital stay. To the best of our knowledge, this is the largest pragmatical and clinical review for the combination therapy of dissolution and endoscopic treatment for bezoars. This review should help physicians to manage bezoars more efficiently.

## 1. Introduction

Bezoars are relatively rare abnormal concretions of indigestible organic material in the gastrointestinal tract, with a reported incidence of 0.07%–0.4% [1–3]. They are classified according to their component materials such as plant and vegetable fiber (phytobezoars), hair (trichobezoars), medication (pharmacobezoars), and milk protein in milk-fed infants (lactobezoars). The most common type of gastrointestinal bezoars is phytobezoars, which occur because of plant materials such as vegetable and fruit, particularly persimmon, fiber, skin, and seed. Bezoars can occur in any part of the gastrointestinal tract, but the stomach is the most common location. They can cause ileus and ulceration because of pressure necrosis and may subsequently lead to gastrointestinal bleeding or perforation. In addition, the gastroparesis is known to be related to the development of bezoars and it might worsen the symptoms of gastrointestinal tract obstruction showing nausea, vomiting, and increase of the risk of complication of ulcerative lesions [4, 5]. Therefore, the removal of these foreign bodies is essential, and although some diospyrobezoars have to be removed surgically, the endoscopic removal method has been increasingly used because of the improvement of various devices. However, because diospyrobezoars may be resistant to endoscopic treatment owing to their stiffness, the dissolution of bezoars using carbonated liquids has been recently described to be an effective treatment option. To date, some reports have emphasized the usefulness of a combination therapy involving dissolution using carbonated liquids [6]; however, no systematic review has been reported about the combination therapy. Therefore, in this study, we reviewed 55 reported cases treated by dissolution therapy combined with or without endoscopic fragmentation introducing 3 additional cases of huge bezoars that were successfully treated using the combination therapy. This pragmatical and clinical review will help physicians to manage bezoars more efficiently.

## 2. Classification of Bezoar

Bezoars are hard masses comprising indigestible food and vegetable fiber or hair found in the gastrointestinal tract, particularly in the stomach. Although the precise incidence of bezoars is unknown, their reported incidence varies from 0.07% to 0.4%. Bezoars are classified into 4 types according to their contents: phytobezoars, trichobezoars, pharmacobezoars, and lactobezoars [7] (Table 1).

### 2.1. Phytobezoar

Phytobezoars are caused by the fibers in food, the most common type. The main food types underlying this condition are celery, grape, prune, pineapple, raisins, and particularly persimmons. The tannin in the food reaction with gastric acid resulted in the polymerization and formation of the mass containing various proteins.

### 2.2. Trichobezoar

Trichobezoars are composed of hair and caused by psychiatric disorders including trichotillomania and trichophagia mainly found in the young women. The hairs form the hair ball with mucous and food in the stomach.

### 2.3. Pharmacobezoar

Pharmacobezoars are caused by medication and its insoluble synthetic compounds, for example, cellulose acetate. The modification on the coating to stabilize the medicine in the stomach using a polymer barrier may lead to the insolubility and be responsible for bezoar formation.

### 2.4. Lactobezoar

Lactobezoars are mainly caused by the undigested milk protein found in milk-fed infants. The number of cases is decreasing; however, the reason is not clarified.

Among these bezoars, phytobezoars are the most common type and the number of cases reported depended on the culture of food intake; they are found especially in the east and west Asian countries where the residents have the habit of eating persimmons. Therefore the reports for the management and therapeutic strategies have been reported from these countries with treating various cases. However, it is obvious that any indigestible materials including not only food but also foreign bodies which can cause mass with mucus and so forth can be the etiology of the bezoar.

## 3. Clinical Symptoms and Management

### 3.1. Symptoms

Bezoars can be found in any age groups depending on the types. However, as the frequency of the phytobezoars is much higher than the other types of bezoar, they tend to be found in the adults (Table 2). Among 55 cases reviewed, other than one case clearly related to the medication (pharmacobezoars), the majority of the cases showed phytobezoar caused by the oral uptake of fruits, green juice, and so forth which contain large amount of fiber or tannin (Table 3). Bezoars can be asymptomatic; however, they often cause ileus, gastric ulcer, Mallory-Weiss syndrome, gastric erosions, and subsequent gastrointestinal bleeding or perforation (Table 2). The majority of the bezoars are found in the stomach; however, some of them move into the small intestine and to colon and might cause intestinal obstruction. Reviewing 55 cases (Table 4), 60% of cases showed complications in their gastrointestinal tract. Therefore, the removal of bezoars is a necessity, particularly if they are huge.

### 3.2. Management

The treatment modalities for bezoars include endoscopic therapy with fragmentation, dissolution using carbonated liquids, medical treatment involving enzymatic dissolution, and surgery [17]. The enzymatic dissolution includes the utilization of papain, cellulose, and so forth. The papain is the enzyme purified from the Papaya which hydrolyzes proteins. The cellulose is the component of green plants and induces cleavage of glycosidic linkage in cellulose which is the component of the bezoars. The surgical removal methodologies are used when the case shows intestinal obstruction, refractory bezoars, and when the endoscopic procedure failed, particularly when caused by persimmons. Recently, less invasive laparoscopic removal method has been used [15, 17]. Endoscopic procedure has been used to fragment the bezoars. Various devices, including snares and forceps, are used to fragment the bezoar and a basket catheter is used to remove the fragmented bezoars; otherwise, these small pieces of bezoars may cause intestinal obstruction. The major limitation of the procedure is, however, since the success of the procedure depends on the stiffness of the bezoars and persimmon phytobezoars, that the majority of the bezoars need multiple sessions which might cause the longer hospital stay and the complications. The dissolution therapy using the carbonated liquids has been reported recently. The first report of dissolution using Coca-Cola was published in 2002 by Ladas et al. [18]. Since then, successful treatments have been documented using various low calorie cola drinks [14, 16, 21, 32]. The mechanisms of the effectiveness have not been elucidated; however, the carbon dioxide bubbles may penetrate the bezoar and make it softer. Since endoscopic therapy or dissolution alone is often time consuming and may lead to the development of intestinal obstruction because of the fragmented, residual bezoars [8], some articles have reported that the combination of endoscopic therapy and dissolution could successfully remove hard and huge bezoars and reduce the period of treatment [9].

## 4. Endoscopic Removal and Dissolution Treatment

With the report of successful treatment of gastric bezoar using endoscopic procedure and carbon oxide fluid, we carefully reviewed a total of 55 patients, including our 3 additional patients, treated by dissolution with (combination therapy) or without endoscopic fragmentation (dissolution monotherapy) [6, 8–14, 16, 19–34] (Table 4) to develop the safe and effective manner for the removal.

We evaluated age, gender, medical history, and associated complications (Table 2). Median age was 67 years (range: 41–91 years). Nineteen patients (35%) had a history of gastric and/or duodenal ulcer, 6 patients (11%) had a history of gastrointestinal surgery, and 18 patients (33%) were complicated by diabetes mellitus. It has been reported that the dysfunction of gastric motility is a risk factor for bezoar formation [4, 5, 35, 36] and the gastroparesis is one of the major reasons of the development of bezoar in Europe and United States [4, 5]. Since it can also cause the ulcerative lesions and worsen the obstructive symptoms, glycemic control, prokinetics, gastroelectrical stimulation, and so forth have been considered to be the management strategies [4]. Older people and those with medical histories and concurrent diseases as described above are more likely to develop bezoars (Table 2).

In addition, to evaluate the usefulness of combination therapy, we retrospectively compared the etiology, total administration amount of carbonated liquids, endoscopic procedure time, length of hospital stay, and outcomes between dissolution and combination therapy (Table 3). There are 21 patients treated by dissolution alone and 34 patients treated by combination therapy. The main cause was persimmon consumption (29/55 patients, 52.7%), followed by green tea or juice consumption containing high levels of tannin, which can sometimes cause a gastric bezoar (2/55 patients, 3.6%) [23]. These etiologies might be affected by the difference of the dietary cultures, since the frequency of the uptake of green tea and persimmon is much higher in Asian countries than Western countries [22].

Over recent years, drinking green juice and eating healthy foods containing tannin have become very popular for fitness. However, we should keep in mind that an excessive consumption of tannin can lead to the formation of a phytobezoar. In the combination group, the average administration amount of carbonated liquids was 4,456 mL (dissolution alone, 3,738 mL; combination therapy, 4,878 mL). This difference might be due to the size of bezoars, since the median size of bezoars was approximately 4.5 cm in dissolution monotherapy, whereas it was 7.0 cm in combination therapy group (overall, 5.4 cm). However, as the limitation of this literature review, the size of each bezoar was estimated as an occupied percentage of the stomach lumen in some patients; therefore, we could not evaluate accurate measurements (Table 3). The average endoscopic procedure time was 52 min for the combination therapy and this data further needs to be evaluated with the data obtained in the larger population study comparing with the endoscopic monotherapy. Interestingly, the average length of hospital stay was 9.3 days in dissolution monotherapy group, whereas it was 4.6 days in combination therapy group (overall 6.4 days). These results may suggest that the combination therapy contributes to cost reduction by shortening the length of hospital stay and reducing the risk of complications related to the endoscopic procedure by shortening the procedure time. This practice would help avoid bleeding, over-tube-associated complications, and intestinal obstruction because of fragmented, residual bezoars.

In terms of outcomes, one patient (Patient 18, 1/21 patients, 5%) underwent surgical removal following the dissolution monotherapy because of intestinal obstruction owing to fragmented bezoars arising because of the procedure using Coca-Cola [8]. For endoscopic monotherapy, Erzurumlu et al. [37] reported a retrospective analysis of 34 cases with gastrointestinal bezoars, with a failure rate of endoscopic therapy being 14.3%. And there was one case (1/34 patients, 2.9%) treated using the combination therapy followed by surgical removal. In this patient (Patient 38; Table 4), the reduced size and softer consistency of the bezoar were apparent following dissolution using Coca-Cola, and combination therapy seems to be successfully performed after giving additional cola orally for 2 days; however, the patient hoped for the surgical removal [13]. Consequently, the combination therapy for gastric bezoars with dissolution and endoscopic fragmentation may be more effective comparing with the monotherapy. The following are the three representative cases recently added from our institute and the procedure present here can help physicians to treat bezoars efficiently.

## 5. Representative Case Presentation

### 5.1. Case 1

A 63-year-old Japanese woman visited a hospital with anorexia and heart burn. She had no history of gastritis or any other disease, except appendicitis. Upper gastrointestinal endoscopy revealed a huge bezoar in the patient's stomach, which was not detected in a 1-year prior examination. The patient was referred to our hospital. Her physical examination revealed upper abdominal tenderness and laboratory results were within normal ranges. The patient reported a habit of drinking green juice extracted from vegetables every day. This juice contained a high volume of catechin, a type of tannin.

**Figure 1:**
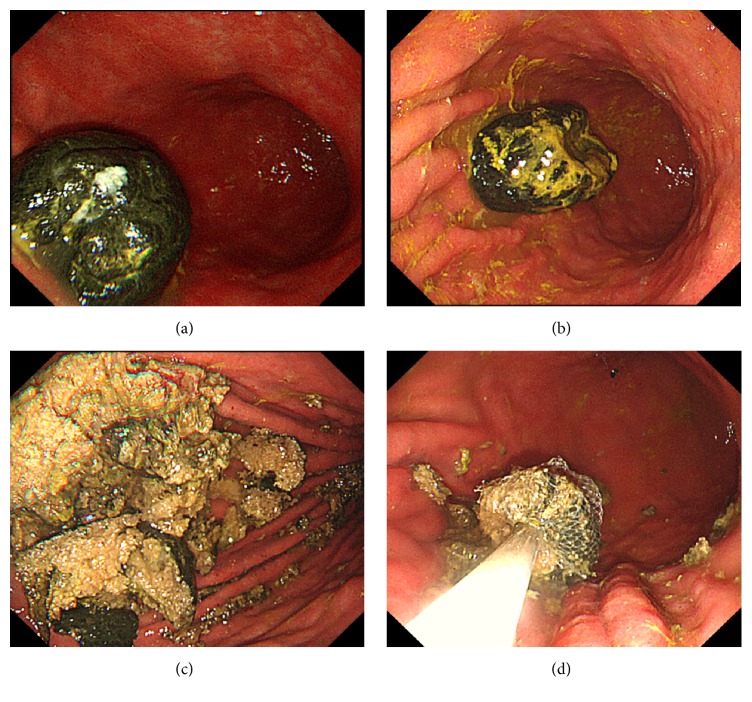
(a) Approximately 5 cm of the bezoar in the stomach before the dissolution therapy. (b) The bezoar showed depression on its surface after the dissolution. (c, d) The bezoar became softer after the dissolution made the endoscopic procedure easier.

Upper gastrointestinal endoscopy revealed a black-colored bezoar measuring approximately 5 cm in the greater curvature of the gastric body, with no ulcers or other complications in the gastrointestinal tract (Figure 1(a)). Next, we attempted an endoscopic removal of the bezoar using biopsy forceps; this approach failed owing to the stiffness of the bezoar. Therefore, to soften the bezoar wall and break it into smaller pieces, we administered 500 mL of Coca-Cola every day for 2 days followed by an endoscopic examination. The bezoar showed reduction of its size especially on its surface (Figure 1(b)) compared to the pretreatment. In addition it was successfully softened following the administration of Coca-Cola, allowing the mass to be easily broken into pieces using biopsy forceps and the snare and it was successfully removed (Figures 1(b)–1(d)). The endoscopic procedure time was approximately 55 min. The patient was discharged on the third day after treatment without complications. A component analysis of the portion of the bezoar removed revealed that it comprised >98% of tannin, indicating that the green juice was probably the cause.

### 5.2. Case 2

A 70-year-old Japanese man underwent annual gastrointestinal endoscopy as a follow-up screening process after a distal gastrectomy performed 15 years prior for gastric cancer. He was referred to our hospital because the upper gastrointestinal endoscopy revealed a huge bezoar in his remnant stomach. He had no symptoms and laboratory results revealed no abnormal findings upon admission. However, he had consumed 2-3 persimmons every day for 3-4 months from autumn to winter in the previous year.

**Figure 2:**
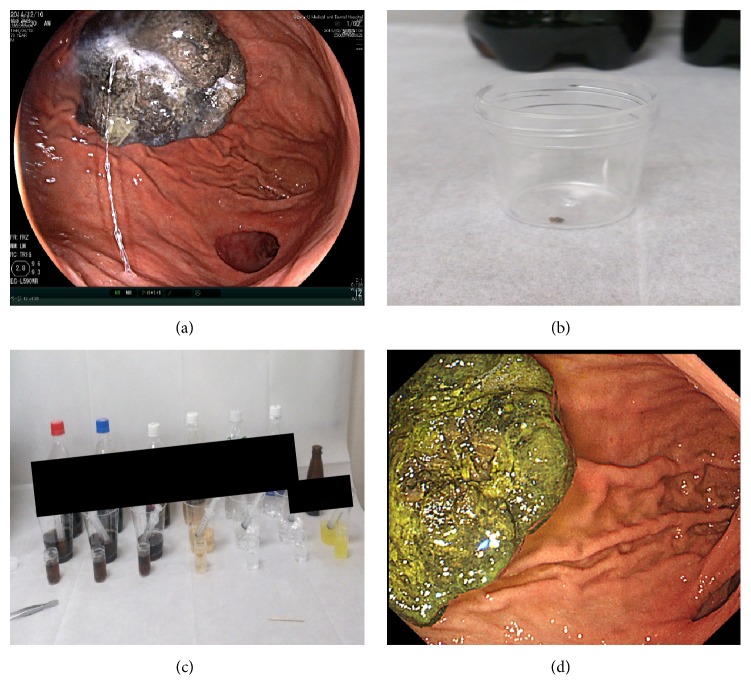
(a) Approximately 5 cm of the black bezoar in the remnant stomach before the dissolution therapy. (b, c) Small pieces of the bezoar had their solubility examined in seven different carbonated liquids. (d) The size and color of bezoar after the dissolution therapy using the liquid showed the highest dissolution effect in (c).

There were no ulcers or other complications in his remnant stomach, and a black-colored bezoar measuring approximately 5 cm was observed in the greater curvature of the upper gastric body (Figure 2(a)). The stiffness of the bezoar resulted in difficulty in breaking and removing the bezoar using biopsy forceps within 1 h. Therefore, we planned dissolution using carbonated liquids prior to the second removal attempt. To examine whether different carbonated liquids exerted different effects on solubility, we removed small pieces of the bezoar and placed individual pieces in seven different carbonated juices (Figures 2(b) and 2(c)). Of these, Juice X showed the best solubility in terms of breaking the piece of the bezoar within a short period of time. Consequently, we gave the patient 500 mL of Juice X each day for 2 days. Gastrointestinal endoscopy performed after 2 days of Juice X administration showed that the bezoar had become softer (Figure 2(d)). The mass was easily broken into small pieces using a polypectomy snare and subsequently removed using a collection net. The endoscopic procedure time for this method was approximately 28 min. Component analysis showed that this bezoar comprised >98% of tannin, probably because of the uptake of too many persimmons in the previous year. On the next day, oral feeding was commenced and the posttreatment period remained uneventful. The patient was discharged on the second day after treatment without complications.

### 5.3. Case 3

**Figure 3:**
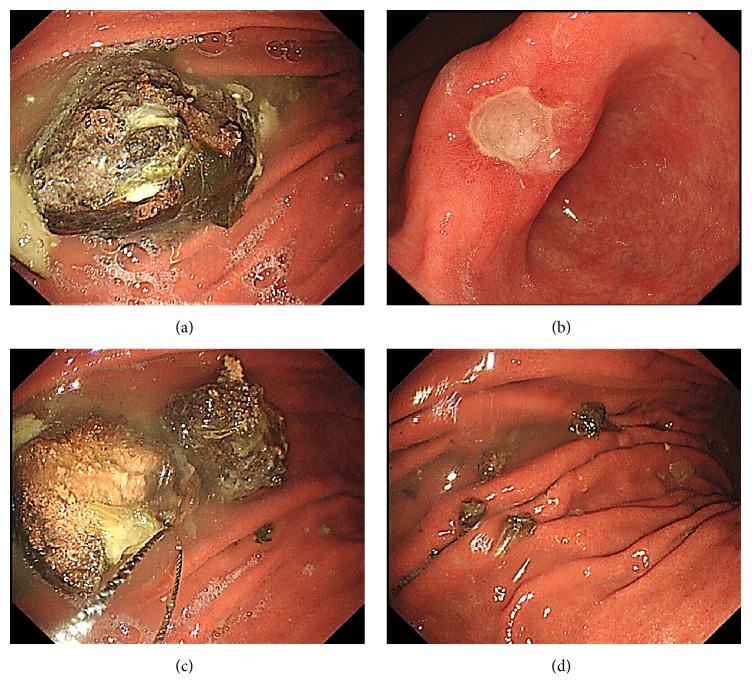
(a, b) Approximately 4 cm of the bezoar with a gastric ulcer (A2 stage) in the lesser curvature. (c) The polypectomy snare and gripping forceps were utilized for fractioning. (d) Successful removal of the bezoar.

An 80-year-old Japanese man underwent endoscopy to investigate symptoms of epigastric discomfort over the previous month. Examination revealed the existence of a huge bezoar and he was referred to our hospital. His physical examination revealed mild epigastric pain and tenderness and laboratory results were within normal ranges. He also had consumed persimmons over the previous year. Endoscopy showed a hard, black-colored bezoar measuring approximately 4 cm in the stomach complicated with a gastric ulcer (A2 stage) in the lesser curvature of the gastric angular area (Figures 3(a) and 3(b)). We collected some broken pieces to determine the most appropriate carbonated juice for dissolution as described for case 2. There was no significant difference between the juices in terms of softening the bezoar; therefore, we used the same protocol as with case 2. The patient was given 500 mL of Juice X every day for 2 days, followed by an endoscopic removal. The polypectomy snare was used as in the previous cases, and gripping forceps were also required for this case (Figure 3(c)). However, the bezoar was successfully broken into small pieces and completely removed using a collection net (Figure 3(d)). The endoscopic procedure time was approximately 19 min. Component analysis showed >98% of tannin in this bezoar, probably because of the uptake of too many persimmons over the previous year. The posttreatment period was uneventful and he was discharged on the next day.

## 6. Discussion

Bezoars are hard masses comprising indigestible food and vegetable fiber or hair found particularly in the stomach. They are classified into 4 types according to their contents (Table 1). Phytobezoars are the most common type. The main food types underlying this condition are various fruits and vegetables, particularly persimmons (as with cases 2 and 3 in the present study). Bezoars often cause ileus, gastric ulcer, and subsequent gastrointestinal bleeding or perforation occasionally complicated with the gastroparesis due to the systemic diseases, such as diabetes mellitus, and preexistence of the gastric outlet obstruction. Therefore, the treatment of etiologies and removal of bezoars are essential, particularly if they are huge. The treatment modalities for bezoars include (1) glycemic control, antiemetics, and prokinetics for gastroparesis [4]; (2) endoscopic therapy with fragmentation, dissolution using carbonated liquids, and medical treatment involving enzymatic dissolution; and (3) surgery including laparoscopic procedure [17]. Ladas et al. reported the first report of dissolution using carbonated drink in 2002 [18] and successful treatments have been documented using various drinks [14, 16, 21, 32]. Although in some cases with hard bezoars surgical intervention is needed, however, the combination of endoscopic procedure and carbonated drinks is showing safer and more effective results than endoscopic monotherapy [15]. The advantage of the combination is that the carbonated drink can make bezoar softer and endoscopic procedure can help in fragmentation and removal of the pieces preventing the intestinal obstruction [8]. Recent article has reported that the combination of endoscopic therapy and dissolution could successfully remove hard and huge bezoars and reduce the period of treatment [9].

In this study, we reviewed a total of 55 patients, including our 3 additional patients, treated by dissolution with or without endoscopic fragmentation [6, 8–14, 16, 19–34] (Table 4). In terms of efficacy and outcomes, the average length of hospital stay was shorter in the group treated with combination procedure of dissolution by carbonated liquid and endoscopic fragmentation and removal. In addition, only one case showed failure of removal and underwent surgical removal following the combination therapy due to the failure of removal (Table 4), while 14.3% of cases failed to be treated with endoscopic monotherapy. These results may suggest that the combination therapy contributes to cost reduction by shortening the length of hospital stay and reducing the risk of complications related to the endoscopic procedure by shortening the procedure time. Consequently, the combination therapy for gastric bezoars with dissolution and endoscopic fragmentation may be more effective comparing with the monotherapy.

In our representative 3 cases, we investigated the solubility of bezoars by testing various carbonated liquids* in vitro*. Small pieces of bezoar were laced into individual tubes containing different carbonated liquids. In case 2, Juice X had the most superior solubility among the liquids tested. The bezoar was softened and fragmented easily following the administration of Juice X at a dose of 500 mL every day for 2 days. Diospyrobezoars are often resistant to dissolution because of their stiffness and are usually endoscopically or surgically removed. In the present study, the diospyrobezoars found in case 3 were treated using the combination therapy. However, the contribution of the endoscopic procedure is relatively better using various devices. This experience suggests that it may be effective and reasonable to determine the therapeutic strategy for each phytobezoar to investigate the therapeutic effect of dissolution therapy by several carbonated liquids in advance. Differences between liquids reflect differences in the concentration of carbon dioxide, pH value, and contents such as sugar, thereby contributing to differences in solubility, although it is not disclosed from the company. It is reasonable to determine the best carbonated liquid prior to the procedure to reduce the hospital stay, the procedure time, and risks of the procedure, leading to the safe and effective removal. Further cases are necessary to be studied as randomized prospective study.

## 7. Conclusion

We carefully reviewed 55 cases including our 3 cases of gastrointestinal bezoars to summarize the knowledge and data for developing the safe and efficient therapeutic strategy. The data showed the efficiency in the combination of dissolution therapy using carbonated liquid and endoscopic procedure which resulted in the shorter period of the hospital stay. Therefore the selection of the carbonated liquid prior to the intervention will further help patients to be treated in safe and efficient manner by reducing the endoscopic procedure time. In conclusion, the results of our literature review should help physicians to treat gastric bezoars in a much more effective manner.

## Figures and Tables

**Table 1:** Classification of bezoar and component.

Classification	Component
Phytobezoars	Plant and vegetable fiber
Trichobezoars	Hair ball
Pharmacobezoars	Conglomerations of medications
Lactobezoars	Undigested milk protein

**Table 2:** Clinical characteristics of patients (*n* = 55).

Median age (yr, range)	66.1 (41–91)
Gender (*n*, male : female)	32 : 23
Medical history	
Diabetes mellitus	18 (32.7%)
Gastric and/or duodenal ulcer	19 (34.5%)
Gastrointestinal tract surgery	6 (10.9%)
Complication	
Gastric ulcer	26 (47.2%)
Ileus	3 (5.5%)
Mallory-Weiss syndrome	1 (3.6%)
Gastric erosion	1 (3.6%)

**Table 3:** Comparison of clinical characteristics between dissolution therapy and combination therapy groups.

	Dissolution therapy group (*n* = 21)	Combination therapy group (*n* = 34)
Etiology		
Persimmon	8 (38%)	21 (62%)
Green tea or juice	0	2 (6%)
Medication	0	1 (3%)
N/A	13 (62%)	10 (29%)
Total administration amount of carbonated drinks (mL)	3,738	4,878
Endoscopic procedure time (min)	—	52
Length of hospital stay (day)	9.3	4.6
Outcome		
Success	20 (95%)	33 (97%)
Failure	1 (5%, surgically removed)	1 (3%, surgically removed)
Size of bezoar (median, cm)	4.5	7

**Table 4:** Summary of 55 patients treated by the dissolution therapy in combination with or without endoscopic fragmentation.

Number	Age	Sex	Past history	Combination with endoscopic removal	Administered carbonated drinks	Administration method	Etiology (type of bezoar)	Size (cm or % of lumen)	Total amount (mL)	Single dose (mL)	The number of administration treatment (times)	Endoscopic procedure time (min)	The length of hospital stay (day)	Complication	Outcome	Reference
1	57	M	None	−	Coca-Cola®	Nasogastric tube	N/A (phytobezoar)	N/A	3,000	3,000	1	N/A	1	None	Success	[7]
2	N/A	M	Diabetes mellitus	−	Coca-Cola Light®	Nasogastric tube	N/A (phytobezoar)	N/A	3,000	3,000	1	N/A	1	None	Success	[7]
3	N/A	M	Diabetes mellitus	−	Coca-Cola Light	Nasogastric tube	N/A (phytobezoar)	N/A	3,000	3,000	1	N/A	1	None	Success	[7]
4	N/A	M	Diabetes mellitus	−	Coca-Cola Light	Nasogastric tube	N/A (phytobezoar)	N/A	3,000	3,000	1	N/A	1	None	Success	[7]
5	N/A	F	Diabetes mellitus	−	Coca-Cola Light	Nasogastric tube	N/A (phytobezoar)	N/A	3,000	3,000	1	N/A	1	None	Success	[7]
6	52	F	None	−	Coca-Cola	Nasogastric tube	Persimmon	4 cm	3,000	3,000	1	N/A	1	Ileus	Success	[8]
7	42	M	None	−	Coca-Cola	Endoscopy	N/A (phytobezoar)	N/A	120	120	1	N/A	1	None	Success	[9]
8	41	M	Diabetes mellitus	−	Coca-Cola Light	Peroral and endoscopy	N/A (phytobezoar)	N/A	10,120	500	7	N/A	3	None	Success	[10]
9	55	M	None	−	Coca-Cola	Peroral	N/A (phytobezoar)	N/A	N/A	800	N/A	N/A	60	Gastric ulcer	Success	[11]
10	73	M	None	−	Coca-Cola	Peroral	N/A (phytobezoar)	N/A	N/A	800	N/A	N/A	60	None	Success	[11]
11	83	M	None	−	Coca-Cola	Peroral	Persimmon	4 cm	3,500	500	7	N/A	7	Gastric ulcer	Success	[12]
12	73	M	Hypereosinophilic syndrome	−	Coca-Cola	Peroral and endoscopy	Persimmon	N/A	6,000	500	14	N/A	8	Ileus	Success	[13]
13	63	M	Duodenal ulcer	−	Coca-Cola	Peroral, ERCP cannula, nasogastric tube	Persimmon	5 cm	N/A	1,000	39	N/A	22	Gastric ulcer	Success	[14]
14	69	F	None	−	Coca-Cola	Peroral	N/A (phytobezoar)	Above 50%	3,000	3,000	1	N/A	2	Gastric ulcer	Success	[15]
15	62	F	Diabetes mellitus	−	Coca-Cola	Nasogastric tube	N/A (phytobezoar)	Above 50%	3,000	3,000	1	N/A	2	Gastric ulcer	Success	[15]
16	49	F	Diabetes mellitus and gastric ulcer	−	Coca-Cola	Nasogastric tube	N/A (phytobezoar)	Below 50%	3,000	3,000	1	N/A	3	None	Success	[15]
17	51	F	Diabetes mellitus	−	Coca-Cola	Nasogastric tube	N/A (phytobezoar)	Below 50%	3,000	3,000	1	N/A	2	Gastric ulcer	Success	[15]
18	75	M	None	−	Coca-Cola	Peroral	Persimmon	N/A	1,800	360	5	N/A	6	Ileus	Surgically removed	[14]
19	83	F	Diabetes mellitus	−	Coca-Cola, Coca-Cola ZERO®	Peroral	Persimmon	5 cm	N/A	1,500	N/A	N/A	8	Gastric ulcer and Mallory-Weiss syndrome	Success	[16]
20	91	F	Brain tumor	−	Carbonated water	Nasogastric tube	Persimmon	5 cm	6,000	500	3	N/A	3	Gastric ulcer	Success	[4]
21	79	F	Rectal cancer, pancreatic cancer, duodenal cancer	−	Carbonated water	Nasogastric tube	Persimmon	4 cm	6,000	500	3	N/A	3	Gastric ulcer	Success	[4]
22	82	M	Diabetes mellitus, hypertension, pulmonary fibrosis	+	Diet Pepsi-Cola®	Peroral	Persimmon	N/A	4,000	1,000	4	N/A	5	Gastric ulcer	Success	[17]
23	50	M	Gastric ulcer	+	Diet Pepsi-Cola	Peroral	Persimmon	7 cm	1,050	350	3	N/A	1	Gastric ulcer	Success	[18]
24	67	M	Gastric cancer, diabetes mellitus, hypertension, COPD	+	Coca-Cola	Nasogastric tube	Medications	N/A	N/A	200	N/A	N/A	3	None	Success	[19]
25	87	F	Angina	+	Coca-Cola	Nasogastric tube	Persimmon	7 cm	1,950	650	3	N/A	3	Gastric ulcer and incarceration of stomach vestibular part	Success	[20]
26	48	M	Duodenal ulcer	+	Coca-Cola	Peroral	N/A (phytobezoar)	Below 50%	6,000	3,000	N/A	8	3	None	Success	[15]
27	57	F	Gastric ulcer	+	Coca-Cola	Nasogastric tube	Persimmon	Above 50%	6,000	3,000	N/A	45	3	None	Success	[15]
28	71	M	Gastric ulcer	+	Coca-Cola	Peroral	Persimmon	Below 50%	6,000	3,000	N/A	14	2	None	Success	[15]
29	65	F	Gastric ulcer and hypertension	+	Coca-Cola	Nasogastric tube	Persimmon	Above 50%	6,000	3,000	N/A	44	3	None	Success	[15]
30	61	F	Diabetes mellitus and gastric ulcer	+	Coca-Cola	Peroral	Persimmon	Below 50%	6,000	3,000	N/A	62	4	None	Success	[15]
31	57	F	Gastric ulcer	+	Coca-Cola	Nasogastric tube	Persimmon	Below 50%	6,000	3,000	N/A	42	3	None	Success	[15]
32	67	F	Diabetes mellitus	+	Coca-Cola	Nasogastric tube	Persimmon	Below 50%	6,000	3,000	N/A	50	7	Gastric ulcer	Success	[15]
33	63	M	Gastric ulcer and hypertension	+	Coca-Cola	Nasogastric tube	N/A (phytobezoar)	Above 50%	6,000	3,000	N/A	30	3	None	Success	[15]
34	78	M	Diabetes mellitus	+	Coca-Cola	Nasogastric tube	Persimmon	Above 50%	9,000	3,000	N/A	52	3	Gastric ulcer	Success	[15]
35	75	F	Diabetes mellitus	+	Coca-Cola	Peroral	Persimmon	Below 50%	6,000	3,000	N/A	40	5	None	Success	[15]
36	54	F	Hypertension	+	Coca-Cola	Peroral	Persimmon	Below 50%	9,000	3,000	N/A	58	5	Gastric ulcer	Success	[15]
37	61	F	None	+	Coca-Cola	Peroral	Persimmon	Below 50%	6,000	3,000	N/A	62	5	Gastric ulcer	Success	[15]
38	63	M	Gastric ulcer	+	Coca-Cola	Peroral	Persimmon	Above 50%	6,000	3,000	N/A	72	3	None	Surgically removed	[15]
39	78	M	Diabetes mellitus, gastric ulcer, hypertension	+	Diet Coca-Cola®	Peroral	N/A	5 cm	4,000	1,000	4	N/A	3	Gastric ulcer	Success	[21]
40	76	F	Gastric cancer	+	Coca-Cola	Peroral and ERCP cannula	N/A (phytobezoar)	N/A	N/A	N/A	1	N/A	1	Gastric erosion	Success	[22]
41	74	M	Gastric cancer	+	Coca-Cola	Peroral	N/A (phytobezoar)	7 cm	1,400	700	2	N/A	2	Anastomotic ulcer	Success	[23]
42	66	F	None	+	Coca-Cola	Peroral	Persimmon	7.8 cm	21,000	1,500	14	N/A	16	Gastric ulcer	Success	[16]
43	69	M	None	+	Coca-Cola	Peroral	Persimmon	N/A	3,000	1,000	3	180	3	Gastric ulcer	Success	[24]
44	70	M	Diabetes mellitus	+	Coca-Cola	Peroral	N/A	N/A	5,550	1,850	3	60	3	Gastric ulcer	Success	[24]
45	52	M	Duodenal ulcer	+	Coca-Cola	Peroral	N/A	N/A	4,500	4,500	2	60	2	Gastric ulcer	Success	[24]
46	78	M	Diabetes mellitus and gastric ulcer	+	Coca-Cola	Peroral	Persimmon	N/A	4,000	1,000	4	N/A	2	Gastric ulcer	Success	[25]
47	57	F	Gastric ulcer	+	Coca-Cola	Endoscopy	Persimmon	7 cm	500	500	2	N/A	6	None	Success	[26]
48	77	M	Gastric ulcer	+	Coca-Cola	Peroral and endoscopy	Green tea	9 cm	N/A	1,500	3	N/A	6	None	Success	[27]
49	68	M	Rectal perforation, gastric cancer, acute pancreatitis, acute cholecystitis	+	Coca-Cola	Nasogastric tube	N/A (phytobezoar)	5 cm	3,000	3,000	2	60	3	None	Success	[28]
50	57	F	Gastric ulcer	+	Coca-Cola	Endoscopy	N/A	N/A	500	500	1	N/A	4	None	Success	[29]
51	74	M	Diabetes mellitus, Cerebral infarct	+	Coca-Cola	Ileus tube and endoscopy	N/A	7 cm	N/A	100	8	N/A	29	Ileus and gastric ulcer	Success	[30]
52	59	M	Epilepsy, duodenal perforation	+	Coca-Cola	Peroral	Persimmon	6.5 cm	N/A	1,000	5	N/A	5	Gastric ulcer	Success	[31]
53	63	F	Appendectomy	+	Ginger Ale	Peroral	Green tea	5 cm	1,000	500	1	55	5	None	Success	Our case
54	70	M	Total gastrectomy	+	Ginger Ale	Peroral	Persimmon	5 cm	1,000	500	1	28	4	None	Success	Our case
55	80	M	None	+	Ginger Ale	Peroral	Persimmon	4 cm	1,000	500	1	19	3	Gastric ulcer	Success	Our case
